# Comparative Evaluation of Vasorelaxant and Antiplatelet Activity of Two Plant-Derived Benzoquinones: Rapanone and Embelin

**DOI:** 10.3390/molecules30040845

**Published:** 2025-02-12

**Authors:** Dagmara Wróbel-Biedrawa, Monika Kubacka, Magdalena Kotańska, Marek Bednarski, Karolina Grabowska, Irma Podolak

**Affiliations:** 1 Department of Pharmacognosy, Medical College, Jagiellonian University, Medyczna 9, 30-688 Cracow, Poland; dagmara.wrobel-biedrawa@uj.edu.pl (D.W.-B.); karolina1.grabowska@uj.edu.pl (K.G.); 2 Department of Pharmacodynamics, Medical College, Jagiellonian University, Medyczna 9, 30-688 Cracow, Poland; monika.kubacka@uj.edu.pl; 3 Department of Pharmacological Screening, Medical College, Jagiellonian University, Medyczna 9, 30-688 Cracow, Poland; magda.dudek@uj.edu.pl (M.K.); marek.bednarski@uj.edu.pl (M.B.)

**Keywords:** rapanone, embelin, vasorelaxant, antiplatelet, anti-aggregation, cardiovascular diseases, *Ardisia crenata*, *Lysimachia punctata*

## Abstract

Vasorelaxant and antiplatelet agents play an important role in preventing and combating endothelial dysfunction, atherosclerosis and a plethora of associated cardiovascular diseases (CVDs). CVDs are the leading cause of death worldwide and nowadays occur not only in developed but also in developing societies. They include, among others, coronary heart disease, cerebrovascular disease and peripheral artery disease. Due to their high prevalence, it is important to seek efficient preventive measures, such as lifestyle changes and the implementation of appropriate herbal dietary supplementation and treatment alternatives. Plant-derived quinones have recently drawn researchers’ attention due to their interesting biological potential. Embelin and rapanone are two plant-derived benzoquinones with anti-inflammatory and antioxidant properties. Embelin has already been shown to have vasorelaxant and antiplatelet activity, but little is known about rapanone in the context of CVDs. Therefore, we decided to comparatively evaluate their activity in a specially designed experimental protocol. Following the isolation of both benzoquinones from plant sources (rapanone from *Ardisia crenata* leaves; embelin from *Lysimachia punctata* roots), their effects were comparatively assessed in a biofunctional study on isolated rat aorta (precontracted with phenylephrine) and in vitro on platelet aggregation. Both benzoquinones showed 50% vasorelaxation in an NO-dependent manner. Interestingly, rapanone was slightly more effective as an antiplatelet agent than embelin. The antiplatelet effect of both benzoquinones was specific, as no cytotoxicity towards platelets was observed at the concentrations tested. This is the first report on the vasorelaxant and antiplatelet activity of rapanone.

## 1. Introduction

Endothelial dysfunction refers to changes within blood vessels that are characterized by reduced vasorelaxation, a procoagulant tendency and inflammatory changes [1]. It is involved in the pathogenesis of atherosclerosis and most cardiovascular diseases (CVDs). They are represented by coronary artery disease (endothelial dysfunction has recently been classified as a type of non-obstructive coronary artery disease), cerebrovascular disease or peripheral artery disease, and account for 32% of all deaths worldwide in 2019 [2,3]. In the pathophysiology of different CVDs, the development of atherosclerosis is a common denominator [2]. Furthermore, as observed, among the modifiable risk factors for the development of CVDs, high blood pressure (put simply, the result of an imbalance between vasorelaxant and vasoconstrictor agents) is considered the factor with the strongest evidence of causality [4]. The main endogenous vasodilator released by the endothelium is nitric oxide (NO). Moreover, its activity is not limited to vasorelaxation, as it exhibits anti-aggregative and anti-adhesive effects against platelets and anti-proliferative activity on smooth muscle. Consequently, NO is an important regulator of normal endothelial function as it reduces inflammation, oxidation and platelet activation. Any deficit in NO levels leads to endothelial dysfunction and can further initiate various cardiovascular disorders. Such a dysfunctional endothelium, with unstable, highly inflamed atherosclerotic plaques, is easily damaged, exposing thrombogenic factors, smooth muscles and proteoglycans to platelets from the bloodstream. Furthermore, the exposed, positively charged collagen from the cover of the damaged plaque attracts platelets and is one of the factors that activates them to form a plug, which can initiate further steps in CVD pathogenesis [5]. Trauma or damage to the atherosclerotic plaque leads to the exposure of collagen, which is found in abundance in the subendothelium. It is a potent platelet agonist that has affinity for glycoprotein VI (GPVI) and α2β1 integrin (GPIa/IIa) receptors. GPVI receptor stimulation results in the protein tyrosine phosphorylation cascade. This then activates phospholipase Cγ (PLCγ), phosphoinositide 3-kinases (PI3K) and small G proteins, leading to intracellular Ca^2+^ mobilization in platelets and the subsequent exposure of procoagulation sites. Consequently, thrombin is generated and phospholipase A_2_ is activated. Ca^2+^- and diacylglycerol-regulated guanine nucleotide exchange factor I (Cal-DAG-GEFI) is a critical factor, along with Ca^2+^ sensor activity, initiating the first wave of activation of the small guanosine triphosphatase Rap1 (GTPase Rap1) and causing the first wave of thromboxane A_2_ (TXA_2_) synthesis. Protein kinase C (PKC) is then stimulated, and platelet agonists adenosine diphosphate (ADP) and serotonin (5-HT) are secreted. This leads to the amplification of the aggregation signal and the activation of the GPIIb/IIIa receptor complex, and then to the exposure of fibrinogen binding sites and ultimately platelet aggregation. As a result thromboembolic disorders such as ischemic stroke or myocardial infarction may occur. This is why antiplatelet agents play an important role in their prevention. 

It seems logical, therefore, that improving endothelial function, protecting and strengthening blood vessels, reducing inflammation and stabilizing plaques, together with antiplatelet effects and the normalization of blood pressure, could be an efficient line of defence against various CV disorders, e.g., hypertension, coronary or peripheral artery disease and infarction.

Plants remain an important source of medicinally active compounds, and it is estimated that approximately 40% of currently used small-molecule drugs worldwide are compounds of natural origin [6]. In terms of CVD therapy and prevention, of special value is a pleiotropic mode of action, meaning that a given compound has more than one beneficial effect on the cardiovascular system. Interestingly, many natural-derived substances show multiple effects corresponding to the characteristics of endothelial dysfunction, atherosclerosis and their consequences. One such example is statins, which are well-known drugs of natural origin conventionally used in CVD therapy and prevention. The first statins known (e.g., lovastatin) were discovered as products of some members of the fungal genus *Aspergillus* [7]. Lovastatin (under the name of monacolin K) is found also in red yeast rice, which is white rice (*Oryza sativa*) fermented by *Monascus* sp. [8].

There are also examples of plant-derived compounds with beneficial health effects in the prevention and/or therapy of cardiovascular disorders, such as resveratrol. Many studies have confirmed that this compound of polyphenolic structure, identified in the fruits of *Vitis vinifera* but distributed in other plant species as well (e.g., *Reynoutria*), has potent antioxidant, anti-inflammatory and anti-atherosclerotic effects [9]. Another example is microalgae-derived astaxanthin, xanthophyll carotenoid. Apart from demonstrated for resveratrol activities, it also regulates the lipid profile and has been found to be an antiplatelet fibrinolytic agent in vivo [10,11]. Of interest also are tanshinones, diterpenoid structures with an *o*-quinone moiety derived from the roots of *Salvia miltiorrhiza*, which have recently attracted the attention of researchers. Tanshinone IIa has been shown to exhibit cardioprotective, anti-aggregative, anti-inflammatory and antioxidant effects, inhibit atherosclerotic plaque formation and stabilize vulnerable atherosclerotic plaque [12,13].

In general, many different quinones are recognized as highly active compounds with interesting biological potential. A recent review on natural substances with antithrombotic activity, which included nearly 250 structurally diverse compounds, has indicated that 9% were quinone derivatives [14].

**Figure 1:**
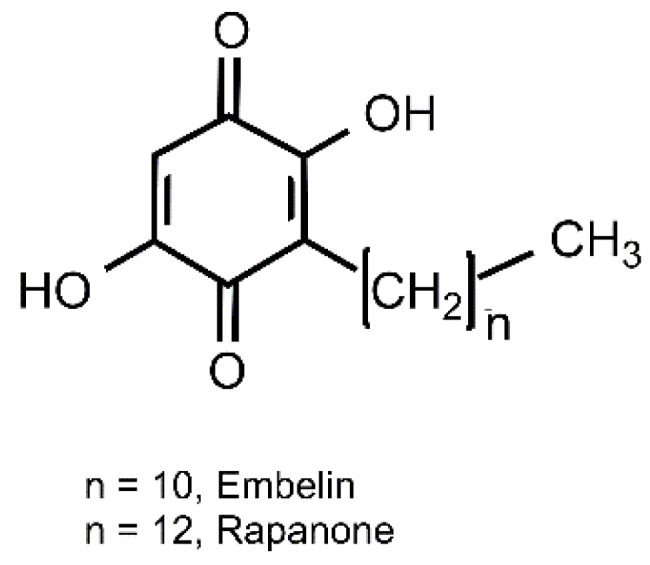
Structure of embelin and rapanone.

Embelin and rapanone, which are the subject of the current study, represent a group of simple alkyl benzoquinones (Figure 1) which differ in the length of the side hydrocarbon chain. They are considered to be chemotaxonomic markers of the Primulaceae family, especially such genera as *Embelia*, *Ardisia*, *Myrsine*, and *Lysimachia* [15,16]. Embelin is a widely studied bioactive compound, with wound-healing, analgesic, contraceptive and cytotoxic properties and chromosome X-linked inhibitor of apoptosis protein (XIAP)-inhibiting anticancer activity (for a review, see [17]). Several reports have confirmed its anti-inflammatory and antioxidant effects [18,19,20]. Furthermore, it has been defined as a vasorelaxant substance and one of the active constituents of *E. ribes* extract, responsible for its anti-hypertensive potential in rats [21]. Together with the data on its antiplatelet activity in vitro and in mice [22], this benzoquinone seems to be an attractive lead compound for research in terms of its potential against CVDs. Rapanone, in contrast, is much less well studied, even though it predominates in some plant sources, such as, for example, *Ardisia crenata*. Scarce data on its pharmacological profile include anti-inflammatory and antioxidant effects [19,23,24]. Our team has been comparatively evaluating the activity of rapanone in relation to embelin for a couple of years. Quite often, slight differences in the structure of compounds can significantly affect their activity; in one of the classic examples, alkaloids vincristine and vinblastine differ in just one constituent. In our studies on embelin and rapanone, despite their high structural similarity, we also found some interesting differences between these two compounds, e.g., in the selectivity of their cytotoxic potential [19,24]. Thus, in view of the above-mentioned activity of rapanone’s structural homologue–embelin, and taking into account that the two compounds usually co-exist in plant sources, we decided to comparatively assess the effects of both benzoquinones on vasorelaxation and antiplatelet activity in in vitro models.

## 2. Results

### 2.1. Effect Induced by Rapanone and Embelin in Rat Aorta Precontracted with Phenylephrine

**Figure 2:**
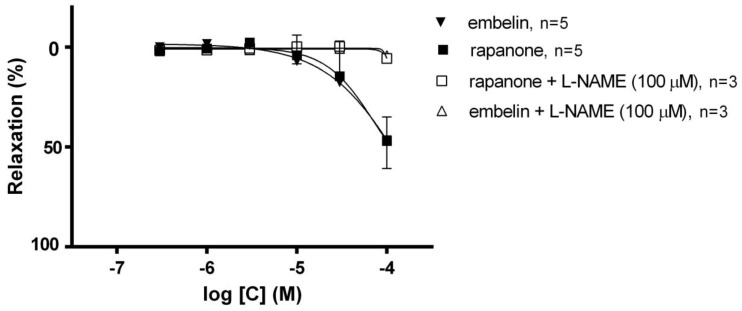
Effect of embelin (▼) and rapanone (■) in endothelium-intact rat aortic rings precontracted with phenylephrine. Effect of L-NAME on embelin (Δ)- and rapanone (□)-induced relaxation in phenylephrine-precontracted endothelium-intact aortic rings. Data are expressed as means ± SD and represent a percentage of relaxation in phenylephrine-induced contraction.

Cumulative concentrations of embelin and rapanone (0.3–100 µM) produced concen-tration-dependent relaxation in phenylephrine (Sigma-Aldrich, Darmstadt, Germany)-precontracted endothelium intact aortic rings, with maximal relaxation of 46.50 ± 5.20% and 49.92 ± 13.8%, respectively, *n* = 5 (Figure 2).

To evaluate the involvement of NO signalling in embelin- and rapanone-induced vasorelaxation, the effects of Nω-Nitro-L-arginine methyl ester hydrochloride (L-NAME (100 µM), Sigma-Aldrich, Darmstadt, Germany), a non-selective nitric oxide synthase (NOS) inhibitor, were tested. Preincubation of endothelium intact aortic rings with L-NAME abolished the vasorelaxation induced by the embelin and rapanone, giving maximal relaxation equal to 4.15 ± 0.09% and 5.65 ± 0.78%, respectively, *n* = 3 (Figure 2).

### 2.2. Effect on Platelet Aggregation In Vitro

**Figure 3:**
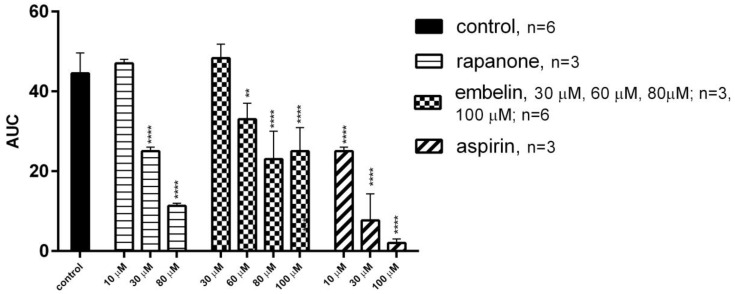
Effects of embelin and rapanone on in vitro whole rat blood aggregation induced by collagen (1.6 µg/mL). Data are expressed as means ± SD; ** *p* < 0.01, **** *p* < 0.0001 vs. control (statistical analysis: one-way ANOVA; post hoc Dunnett’s test). AUC: area under the aggregation curve.

In our study, both rapanone and embelin were able to inhibit collagen (Hyphen-Biomed, Neuville-sur-Oise, France)-induced platelet aggregation (Figure 3). The IC_50_ value for rapanone was equal to 40.2 ± 18.6 µM. Embelin decreased platelet aggregation at the highest concentration tested (80–100 µM) by 51.60% and 56.12%, respectively, and thus the IC_50_ value was not calculated. For comparison, the IC_50_ value for aspirin (Tocris, Abingdon, UK) was equal to 11.60 ± 2.73 μM.

### 2.3. Impact on Platelet Viability

**Figure 4:**
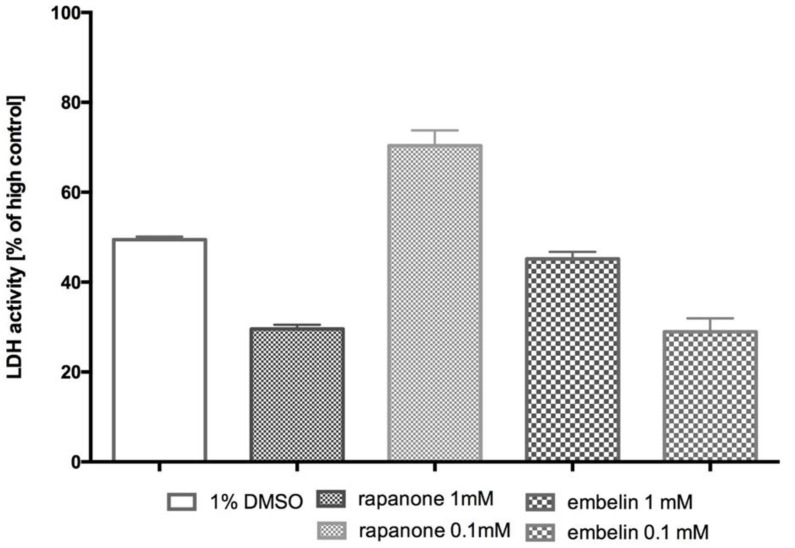
Platelet viability: LDH activity after the incubation of platelets with the tested compound or vehicle (1% DMSO). Mean ± standard deviation (SD), *n* = 2 (the assay was repeated twice in duplicates), Kruskal–Wallis test (ANOVA, *p* = 0.095) and Dunn’s multiple comparison post hoc test (no significance). High control—lysis (medium + lysis solution).

Multiple comparisons did not show significant differences in the lactate dehydrogenase activity (LDH) determined in the control sample vs. in the tested samples (Figure 4). Rapanone at a concentration of 0.1 mM increased LDH activity by approximately 40% compared to the activity determined under the influence of 1% DMSO, which was approximately 70% of the maximum lysis. However, rapanone at a concentration of 1 mM and embelin at both tested concentrations (1 mM and 0.1 mM) did not increase LDH activity, and rapanone at a concentration of 1 mM and embelin at a concentration of 0.1 mM even decreased LDH activity by approximately 40% compared to the activity determined in sample, with only 1% DMSO, and by approximately 70% compared to the activity determined in the sample with maximum lysis.

## 3. Discussion

Vasorelaxant and antiplatelet effects are recognized as important characteristics of preventive and therapeutic agents used in cardiovascular diseases (CVDs), as they are associated with endothelial dysfunction and atherosclerotic changes in blood vessels. CVDs, representing the leading cause of death worldwide, are undoubtedly another consequence of the lifestyle changes that have been introduced over the past centuries. The effects of a diet based on highly/ultra-processed-foods, physical inactivity, tobacco smoking and alcohol consumption can manifest as atherosclerosis, high blood pressure, obesity (especially visceral), hyperglycaemia and raised levels of triglycerides and cholesterol. As the prevention and treatment of CVDs is an unmet need, not only in developed but also developing societies, the search for new, better and safer drugs remains pressing.

Herbal preparations and isolates are widely used worldwide as preventive diet-supplementary agents, as well as therapeutics. They are valued for their complex and harmonized pharmacological effects. Among the promising compounds of plant origin, highly bioactive quinoid structures can be mentioned. Embelin and rapanone represent benzoquinones, which are structurally the simplest group of quinoids. They usually occur together in some species. For the purpose of this study, we isolated the individual benzoquinones from the respective sources, based on our previous experience and results of quantitative studies [25,26]. Hence, rapanone was isolated from *Ardisia crenata* leaves, while embelin was isolated from *Lysimachia punctata* roots.

Despite the structural similarities of the two benzoquinones, we have previously reported some subtle but interesting and important differences in their biological activity [19,24]. In the current study, we aimed to check whether rapanone presents a beneficial profile in the context of CVDs and to compare it to that of embelin, as there are already some results indicating the potential benefits of embelin in CVDs. As was reported, embelin at 50 mg/kg normalized elevated blood pressure and heart rate in high-fat-diet-fed rats [27]. In our study, both benzoquinones showed a moderate vasorelaxant effect on phenylephrine-induced contraction in intact aortic rings taken from rats. At the highest concentration tested, these compounds induced about 50% relaxation. The effect was abolished when L-NAME, the NOS inhibitor, was added to the organ bath, implying that NO is strongly involved in the vasorelaxant effects of rapanone and embelin. Since relaxation is completely blocked in the presence of L-NAME, we can also conclude that endothelial pathways involving prostacyclin or endothelium-dependent hyperpolarizing factor (EDHF) are not involved in the observed effect. However, at this stage of our research, we are not able to identify the exact point(s) in the NO pathway, as they could be due to, e.g., NOS activation or soluble guanylate cyclase (sGC)/K+ channel activation. Nevertheless, in the case of embelin, there are more data available on the pathways that may be involved in its mode of action. As previously reported, it activates NO together with sGC, but the molecular signalling leading to these effects is still unknown [28]. Therefore, further studies are needed to explore the mechanisms of vasorelaxant action of rapanone and embelin. Moreover, in contrast to different quinone derivatives, which revealed the potential to suppress NO formation by inhibiting NOS isoforms, the group of benzoquinones in the different studies did not present any activity, or if they did it was scarcely perceptible [29,30,31].

Another key process involved in the pathogenesis of various CVDs is platelet aggregation induced by unstable plaque. Recently, embelin has been shown to exert antiplatelet effects by blocking protein kinase C (PKC) in aggregation induced by different agonists [22]. Subsequently, it can influence PKB, degranulation (ADP, 5-HT release) or GPIIa/IIIb activation. In addition, the authors reported its antiplatelet and antithrombotic potential in mice. The thrombus formation in the mesenteric microvessels was delayed; however, the tail bleeding time was not significantly changed. The lack of deleterious effects on platelets and the absence of haemostasis disorders confirmed the safety of the compound.

In the current study, we demonstrated the in vitro antiplatelet activity of embelin, but its inhibitory rate was lower (at the highest concentration of 100 µM, aggregation was inhibited at 56.12%) compared to the study of Li et al. (100% of inhibition at 100 µM) [22]. However, this may be due to some methodological differences; for example, we used a higher concentration of collagen (1.6 µg/mL) to initiate aggregation and used rat whole blood, whereas Li’s team used lower collagen concentration (1 µg/mL) and human platelets. Interestingly, rapanone showed an aggregation-inhibitory potential, with IC_50_ = 40.2 ± 18.6 µM, which was stronger than that of embelin but weaker compared to aspirin (IC_50_ = 11.6 ± 2.7 µM). Since our aim was to perform a preliminary study showing the general tendency of rapanone’s activity in comparison to embelin, we chose conditions that are generally accepted in screening, with the concentrations of collagen inducing a strong platelet response. A high concentration of collagen produces an aggregation response involving phospholipase Cγ activation and phosphatidylinositol 4,5-bisphosphate cleavage. Its products then induce Ca^2+^ mobilization and PKC activation. An increase in Ca^2+^ concentration results in morphological changes, degranulation (ADP, 5-HT release), and thrombin-induced aggregation. When a low concentration of collagen is used, the aggregation response is weaker. In this case, an important role is played by a factor amplifying the cascades: thromboxane A_2_. Consequently, the aggregation can be easily blocked or weakened by thromboxane-forming inhibitors, e.g., cyclooxygenase inhibitors.

Since collagen induces platelet aggregation in TXA_2_/ADP/5-HT-dependent manner leading to GPIIb/IIIa activation, the influence of substances that inhibit any of these mechanisms of platelet activation and subsequent aggregation may be detectable. Therefore, collagen provides a general means of assessing platelet function in vitro. As a result, it is not possible at this stage to identify the targets involved in benzoquinone activity. Embelin has previously been shown to block PKC, but more in-depth studies are still needed. We can assume that rapanone should show a similar mode of action, but this needs to be confirmed in further experiments. Nevertheless, this is the first report on its antiplatelet activity. Furthermore, we confirmed that the antiplatelet effect of both benzoquinones was not due to cytotoxic effects on platelets, as platelets viability was not altered compared to the control. Embelin has already been shown to be non-toxic in acute toxicity tests in rodents at doses up to 3 g/kg p.o. Similarly, subchronic administration (10 days) of 10 mg/kg embelin daily was found to be safe. However, some toxic effects were observed in chronic, 6-week trials of embelin at a high dose of 120 mg/kg [32]. Additionally, LD_50_ was estimated as 44 mg/kg when administered i.p. Nevertheless, little is known in this regard about rapanone. According to pharmacokinetic studies of embelin, its bioavailability after oral administration in rats was estimated to be approximately 30% [33]. In our previous studies, we also showed that at lower doses there are some differences between the two benzoquinones in terms of cytotoxic profile, i.e., rapanone generally seemed safer in normal cell lines [19,24]. Still, more results are needed, especially for rapanone, to establish its more comprehensive safety profile.

In addition to our results, it is worth recalling other effects exerted by the benzoquinones in previous studies which seem to be favourable in the context of CVDs. As inflammation has recently gained significance in terms of the initiation and progression of atherosclerosis and other CVDs, the demonstrated anti-inflammatory potential of both studied benzoquinones is undoubtedly an additional benefit. In our previous study, both compounds showed anti-inflammatory activity in the anti-hyaluronidase assay; however, embelin was more potent than both rapanone (IC_50_ = 275.1 vs. 404.7 μg/mL) and the reference used in the test, quercetin (IC_50_ = 517.3 μg/mL) [19]. In addition, embelin has been reported to affect other enzymes, signalling proteins and cytokines, essential in the initiation and propagation of inflammation: interleukin 1β (IL-1β), IL-6, and tumour necrosis factor-α (TNF-α) [18,34,35], nuclear factor kappa-light-chain-enhancer of activated B cells (NF-κB), nuclear factor erythroid 2-related factor 2 (Nrf-2) [18], 5-lipooxygenase (5-LOX), and prostaglandin E_2_ (PGE_2_) synthase-1 [36]. Furthermore, confirmation of this activity in vivo has been shown in several studies [34,37,38,39]. In the case of rapanone, apart from our previous work, there is another report showing its anti-inflammatory properties both in vitro and in vivo [23]. Importantly, these benzoquinones may also regulate the redox potential by affecting endogenous enzymes and due to their own antioxidant potential, which in our previous study was found to be moderate [18,19,40]. As far as we know, oxidative damage has been implicated in the pathogenesis of many conditions, including some CV disorders. For many years, the oxidation of LDL-cholesterol was also thought to be a major trigger for the initiation and progression of atherosclerosis, but in recent years, doubts have been raised about this concept (for a review, see [41]). According to some recent results, native or aggregated LDLs are the main drivers of atherosclerosis. Consequently, the role of oxidative processes and free radicals has become uncertain. Nevertheless, the vasorelaxant and antiplatelet potential of rapanone that was demonstrated for the first time in this study, along with the previously reported anti-inflammatory and antioxidant effect, reveals an interesting scope of activities in terms of the prevention and treatment of CVDs. What is more, *Ardisia crenata*, used in our experiment for rapanone isolation, is also a source of another interesting component, cyclic depsipeptide, FR900359, which has previously revealed an antiplatelet and hypotensive effect in rats [42,43,44]. Consequently, it seems that ardisia leaves may serve as a potential source of compounds with beneficial effect in CVDs.

## 4. Materials and Methods

### 4.1. Standards and Reagents

The materials used included aspirin (Tocris, Abingdon, UK), carbachol (carbamoylcholine chloride, Sigma-Aldrich, Darmstadt, Germany), collagen (Hyphen-Biomed, Neuville-sur-Oise, France), % iodixanol stock solution (OptiPrep density gradient medium, Sigma-Aldrich, Sant Louis, MO, USA), phenylephrine (phenylephrine hydrochloride, Sigma-Aldrich, Darmstadt, Germany), Nω-Nitro-L-arginine methyl ester hydrochloride (L-NAME, Sigma-Aldrich, Darmstadt, Germany) and thiopental (thiopental sodium, Panpharma, Luitré-Dompierre, France). Other chemicals used were obtained from POCH (Polish Chemical Reagents, Warsaw, Poland).

### 4.2. Plant Material

Plant specimens of the white berried variety of *Ardisia crenata* Sims from the Bospremium raising were obtained from a local florist store (Świat Roślin, Cracow, Poland). Botanical identification was performed by Dr. E. Skrzypczak-Pietraszek from the Department of Pharmaceutical Botany, Jagiellonian University Medical College, Cracow, Poland. The voucher specimen (ACRE-W-2017) is deposited at the Department of Pharmacognosy, Jagiellonian University Medical College, Cracow, Poland.

Authenticated roots of *Lysimachia punctata* L. were collected in the Medical Plants Garden (GPS coordinates: latitude 50.011298, longitude 19.994175) at the Faculty of Pharmacy, Jagiellonian University Medical College, Cracow, Poland. The voucher specimen (KFg/2010031) is deposited in the Department of Pharmacognosy, Pharmaceutical Faculty, Medical College, Jagiellonian University, Cracow, Poland.

### 4.3. Extraction and Isolation of Benzoquinones

The isolation of rapanone from leaves of white *A. crenata* Sims and embelin from roots of *L. punctata* L. was carried out according to the procedure described previously [19,24,45]. The purity and identity were checked by the LC-MS method compared to authentic standards, as we previously reported [19].

### 4.4. Animals

In the experiments, male Wistar rats (Krf: (WI) WU), 200–250 g) were used. The animals were housed in constant-temperature facilities and exposed to 12:12 h light/dark cycles, and were maintained on a standard pellet diet with tap water ad libitum. All procedures were conducted according to the Animal Care and Use Committee Guidelines and approved by the Local Ethics Committee of Jagiellonian University in Kraków (resolution no. 148/2015, 24 June 2015).

Ethics statement: The research has been reported to and approved by the Animal Welfare Committee of the Faculty of Pharmacy of Jagiellonian University Medical College (resolution no 1/2023, 19 April 2023).

### 4.5. Effect on Rat Aorta Contracted by Phenylephrine

The thoracic aortas were dissected from rats anesthetized with thiopental sodium (75 mg/kg i.p.). Then, they were placed in a Krebs–Henseleit solution (NaCl 119 mM, KCl 4.7 mM, CaCl_2_ 1.9 mM, MgSO_4_ 1.2 mM, KH_2_PO_4_ 1.2 mM, NaHCO_3_ 25 mM, glucose 11 mM, EDTA 0.05 mM) and cleaned of surrounding fat tissues. Two stainless-steel stirrups were inserted through the lumen of each arterial segment: one stirrup was fixed to the bottom of the chamber and the other to an isometric force-displacement transducer (720MO, DMT, Copenhagen, Denmark). The rings were then put in a 20 mL organ-chamber filled with Krebs–Henseleit solution and gassed with 95%O_2_/5% CO_2_ at 37 °C. The aorta rings were stretched and maintained at the optimal tension of 2 g and allowed to equilibrate for 1 h. Verification of the endothelial integrity was confirmed by the presence of the relaxant response to carbachol (1 μM) on the phenylephrine (1 μM)-contracted vessels. The induction of more than 90% relaxation by carbachol was assumed as evidence that aortic rings were endothelium-intact. After confirmation of the presence of the functional endothelium, vascular tissues were left for 1 h regeneration before any further steps of the experiment.

Then, the effects of the tested compounds on vascular tension were evaluated. Aortic rings with an intact endothelium were contracted with phenylephrine (1 μM) to obtain the maximal response. Once the maximal response to phenylephrine had been evoked, the aortic rings were exposed to cumulative doses of the tested compounds. The responses were recorded.

To determine NO involvement in the relaxant effect of the studied substances, endothelium-intact rings were incubated with a NO synthase inhibitor (L-NAME, 100 μM), and then vascular relaxation was carried out by cumulative additions of the tested compounds at the plateau of the phenylephrine-induced contractions. As L-NAME enhanced phenylephrine-induced contraction, the rings exposed to L-NAME were pre-contracted with phenylephrine 0.6 μM to generate a magnitude of contraction similar to the one found in the rings not exposed to L-NAME.

Relaxations are presented as a percentage of inhibition of the maximal tension achieved with the contractile agent (E_max_ = 100%).

### 4.6. Effect on Platelet Aggregation In Vitro

The in vitro aggregation test was performed using freshly drawn whole rat blood with a Multiplate platelet function analyzer (Roche Diagnostic, Mannheim, Germany) based on the measurements of electric impedance. Blood was obtained from rats’ carotid arteries using a hirudin blood tube (Roche Diagnostic, Mannheim, Germany). Anticoagulated blood (300 μL) was mixed with 300 μL of the prewarmed isotonic saline solution containing the studied compound or vehicle (DMSO 0.1%) and preincubated for 3 min at 37 °C with continuous stirring. Aspirin was utilized as a reference substance. Aggregation was initiated by adding collagen at the final concentration of 1.6 µg/mL. The activated platelet function aggregation process was recorded for 6 min. The Multiplate software v. V2.05 analyzed the area under the curve (AUC) of the clotting process for each measurement and calculated the mean values. Each concentration of studied compounds was tested at least three times. Concentration–inhibition curves were created and analyzed by non-linear curve fitting using GraphPad Prism 6.0 (GraphPad Software Inc., San Diego, CA, USA).

### 4.7. Impact on Platelet Viability

The platelet viability of freshly collected platelet-rich rat plasma was determined according to Kubacka et al. [46]. Whole blood was collected from rats in a tube containing 0.5 mL of PECT medium (0.63 mM Na2CO3, 90 mM disodium edetate, 94 nM prostaglandin E1, and 10 mM theophylline). A density barrier was created by combining 5 mL of 1.320 g/mL 60% iodixanol stock solution (OptiPrep density gradient medium, Sigma-Aldrich, Sant Louis, MO, USA) with 22 mL of diluent (20 mM HEPES-NaOH, pH 7.4, 1 mM disodium edetate, and 0.85% NaCl). For platelet separation, 3 mL of each sample was layered over 5 mL of the density barrier. Samples were centrifuged at 350× g for 15 min at 20 °C [47]. The platelets were then suspended in Barber’s buffer (0.014 M Tris, 0.14 M NaCl, 10 mM glucose; pH 7.4). The number of platelets (1.5–2.0 × 10^8^/mL) used for the test was measured using a spectrophotometer at λ = 800 nm [48]. The dilutions were 20×. The assay was repeated twice in duplicates.

The cytotoxic effect of the tested solutions on blood platelets was evaluated based on the release of lactate dehydrogenase (LDH), according to the instructions given by the kit manufacturer (Merck, Darmstadt, Germany, Cytotoxicity Detection KitPLUS (Cat. No.04744926001)). The time for platelet incubation of the tested compounds with platelets was 10 min.

### 4.8. Data Analysis

All results are expressed as mean ± SD. Concentration–response curves were constructed based on the responses to cumulative concentrations of the studied compounds and analyzed by non-linear curve fitting using GraphPad Prism 6.0 (GraphPad Software Inc., San Diego, CA, USA).

Statistically significant differences between groups were calculated using one-way analysis of variance (ANOVA) and the post hoc Dunnett’s test or Kruskal–Wallis test and Dunn’s multiple comparison post hoc test. The criterion for significance was set at *p* < 0.05.

## 5. Conclusions

In continuation of our studies on the comparative evaluation of rapanone and embelin, two plant-derived benzoquinones, we assessed their vasorelaxant and antiplatelet activities. They were shown to have moderate activity, but given their previously proven anti-inflammatory and antioxidant efficacy, they can be considered as agents with potential health benefits in cardiovascular diseases. The use of collagen was a simple way to assess and compare the overall antiplatelet effects of embelin and rapanone. We are planning to study pathways involved in their antiplatelet effects using different aggregation inducers such as ADP, arachidonic acid, 5-HT, thrombin receptor activating peptide-6 (TRAP-6) or ristocetin. Furthermore, we would like to explore more in-depth mechanisms of their vasorelaxant effect, as well as the interaction between the tested compounds and other components of the plant material in terms of this activity.

## Data Availability

The data presented in this study are available on request from the first author.
